# Serum Vascular Endothelial Growth Factor and C-reactive Protein Serve As Biomarkers of Joint Health in Severe Haemophilia A: A Cross-Sectional Study From India

**DOI:** 10.7759/cureus.117081

**Published:** 2026-09-28

**Authors:** Sukrita Bhattacharjee, Shouriyo Ghosh, Jyoti Shaw, Sudip Roy, Siddhartha S Ray, Maitreyee Bhattacharyya

**Affiliations:** 1 Haematology/Haematology oncology, Chittaranjan National Cancer Institute, Kolkata, IND; 2 Clinical Haematology, Medical College, Kolkata, Kolkata, IND; 3 Clinical Haematology and Cellular Therapies, Tata Medical Center, Kolkata, IND

**Keywords:** abr, crp, fish, functional independence score in haemophilia, haemophilia, haemophilia joint health score, hjhs, joint health, vascular endothelial growth factor, vegf

## Abstract

Objectives

In low- and middle-income countries (LMICs), people living with haemophilia (PWH) remain on on-demand factor replacement therapy and present with haemophilic arthropathy. This two-arm cross-sectional study compared serum vascular endothelial growth factor (VEGF) and C‑reactive protein (CRP) levels between patients with severe haemophilia A and healthy controls and explored their association with clinical indices of joint health.

Materials and methods

We measured serum VEGF and CRP in 50 male patients with severe haemophilia A and 50 age-matched healthy male controls. Joint status was assessed by the Haemophilia Joint Health Score (HJHS), Functional Independence Score in Haemophilia (FISH), and annual bleed rate (ABR). Comparisons used nonparametric tests; diagnostic performance was evaluated using receiver operating characteristic (ROC) curves; and correlations were analysed with Spearman’s rho.

Results

Median age of the patient cohort was 11.5 years (range 4-25 years). Mean VEGF (85.11 ± 9.01 pg/mL) and CRP (1032.58 ± 242.88 pg/mL) levels were significantly higher in PWH than in controls (VEGF 56.9 ± 17.1 pg/mL, CRP 140.26 ± 37.24 pg/mL; both p<0.001). ROC analysis showed fair discrimination between cases and controls for VEGF (area under the curve or AUC 0.792) and CRP (AUC 0.775); optimal cut offs were 68.85 pg/mL for VEGF and 210.03 pg/mL for CRP. VEGF and CRP correlated significantly with FISH and CRP correlated positively with ABR.

Conclusion

Serum VEGF and CRP are elevated in severe haemophilia A and show fair ability to distinguish PWH from healthy controls. Longitudinal studies can help establish the feasibility of these biomarkers in predictive models for joint bleeding.

## Introduction

Haemophilia A is an X-linked recessive bleeding disorder caused by deficiency of clotting factor VIII. It affects one in 5000 live births, and as of 2018, close to 21,000 people have been identified in India [1,2]. Patients with severe haemophilia A (Factor level <1%) have spontaneous and recurrent bleeding in muscles and joints. Repeated bleeding causes synovial inflammation, hypertrophy, and ultimately joint damage. Haemophilic arthropathy (HA) leads to major morbidity in patients with haemophilia [3]. Although certain biochemical markers have been known to be associated with bleeding in haemophilia, reliable predictors of severity of joint damage are not available for severe haemophilia A [4].

People living with haemophilia (PWH) having severe disease present with a variable annual bleeding rates (ABR). Treatment approaches for haemophilia A in low- and middle-income countries (LMICs) like India include on-demand clotting factor concentrate (CFC) replacement therapy along with supportive care. On an average, only 9% of haemophilia A patients receive primary prophylaxis in the country [5,6]. Delays in receiving on-demand factor replacement inevitably leads to haemophilic arthropathy and ultimately poor quality of life. Studies have shown that HA is associated with neovascularisation and synovial proliferation [7,8]. We hypothesized that inflammatory and angiogenic factors such as CRP and vascular endothelial growth factor (VEGF) are elevated in severe haemophilia A and can be correlated with functional measures of joint health.

## Materials and methods

Patients

This was a two-arm cross-sectional observational study performed between August 2021 and December 2024 at a dedicated haemophilia management centre in Eastern India. The study protocol was approved by the Institutional Ethics Committee (ref no. MC/KOL/I EC/NON-SPON/1110/06/2021 dated 11th June 2021) and conforms to the guidelines of the Declaration of Helsinki [9].

Primary and secondary objectives

The primary objective of the study was to compare serum VEGF levels in patients of severe haemophilia A and age-matched healthy male controls. The primary endpoint was to determine the difference in mean serum VEGF level between cases of severe haemophilia A and controls. The secondary objectives were to compare CRP levels in patients and controls and to determine whether any correlation exists between the biomarker levels (VEGF and CRP) and the various indices of joint health such as Haemophilia Joint Health Score (HJHS) [10], Functional Independence score in Haemophilia (FISH) [11] and ABR. The secondary endpoints were to measure the difference in mean CRP level between cases and controls; to determine the correlation between VEGF and CRP levels with HJHS, FISH scores and ABR rates.

Study methods

Patients registered for on-demand and prophylactic factor therapy were recruited in the study. The control group comprised of age-matched healthy male individuals. Exclusion criteria for the study included moderate or mild haemophilia A, haemophilia B, presence of past or current inhibitors or under immune tolerance induction (ITI) therapy, presence of haemophilia-unrelated comorbidities which may affect musculoskeletal system and history of infective events in the preceding three months.

Fifty sequential patients with severe haemophilia A, attending the hospital for on-demand or prophylactic factor replacement were sampled for VEGF and CRP levels prior to the administration of any factor support on the day of the visit. Clinical data including detailed family history, age of onset of bleeding, index and target joints, ABR were collected. The ABR was calculated from the logbook maintained for each patient. HJHS and FISH scores were also recorded after clinical examination.

The 50 healthy male controls were age-matched and underwent blood sampling after ruling out arthropathy and any acute or recent illness from history and physical examination. 

Blood samples (4 ml) were collected in vials from both PWH and the control group by experienced phlebotomist/nurse/healthcare worker with single prick venepuncture. In case of PWH, 23-25-gauge scalp-vein-set needles were used, and pressure was applied to the venepuncture site till bleeding stopped (around five minutes). Samples were immediately transferred to the laboratory. Serum was extracted using standard methods. 

Laboratory estimation of VEGF and CRP levels

Serum VEGF levels were estimated by BioLegend LEGEND MAX™ Human VEGF Sandwich Enzyme-Linked Immunosorbent Assay (ELISA) (BioLegend, Inc, San Diego, US) [12]. VEGF was assayed following the protocol provided with the kit. Recognising the fact that serum VEGF may reflect platelet activation, samples were processed within two hours of collection to minimise ex-vivo release.

Serum CRP was similarly estimated in serum samples by Human C-Reactive Protein ELISA kit (Catalogue no. RAB0096, range 10-20,000 pg/mL, sensitivity <10 pg/mL); manufactured by Sigma-Aldrich (St. Loius, US) as per the procedure described in the kit [13]. The values were reported in pg/mL for consistency with VEGF.

Assessment of HJHS, FISH and ABR

PWH attending the haemophilia clinic were examined and the HJHS was calculated by the attending resident physician and validated by an independent supervising physician. The HJHS score is a seven-item clinical examination score for assessing joint impairment of the six key index joints (elbows, knees, and ankles). Evaluation included assessment and grading of swelling, duration of swelling, muscle atrophy, crepitus on motion, flexion loss, extension loss, joint pain and strength of the knees, ankles and elbows and a global gait score [10]. The highest score is 148, with a higher score indicating worse joint health. The FISH score is established as a performance-based evaluation method to assess functional ability objectively, both before and after therapeutic intervention [11]. Seven activities, including eating, grooming, dressing, chair transfers, squatting, walking, and step-climbing, were evaluated. FISH was assessed as per protocol, omitting 'running' due to space limitations in the clinic; therefore, the maximum score was adjusted to 28 (three domains: self-care, transfers and mobility). ABR was calculated from the history given by the patients or their caregivers and from the logbook maintained for each patient.

Statistical methods

Saboony and colleagues [14] published literature showing a large difference in mean VEGF levels between cases and controls. Therefore, we used Cohen’s convention for standardized effect sizes and applied a large effect size of 0.8. This resulted in a required sample size of 50 patients per group to adequately power the study to detect a true difference in VEGF between the two cohorts.

Demographic data including age, age at diagnosis, ABR, target and index joints were depicted using medians, standard deviation (SD) and range. Mean and standard error of means for VEGF and CRP levels were calculated for the two groups (cases and controls).

For patients, we calculated means for HJHS and FISH scores. For subsequent statistical analyses, we calculated normalized scores for HJHS and FISH scores. This involved dividing the patient's HJHS score by the total HJHS reference standard score and doing the same for the FISH score. This resulted in a ratio of scores for each patient which was then used for analysis.

To compare the means of CRP and VEGF levels between patients and controls, Mann-Whitney U test was applied for this analysis. Subsequently a receiver-operator-characteristic (ROC) curve analysis was done to determine the best cut-offs of VEGF and CRP levels between cases and controls. Area under the curve (AUC) and Youden Index were calculated for analysing the ROC curve results.

For correlations between CRP and VEGF and the indices of joint health (HJHS, ABR, FISH), Kendall’s tau and Spearman rho tests of correlation were applied for the univariate analysis. Bootstrapping was done for all the tests. All tests were done using IBM SPSS Statistics for Windows, Version 31 (Released 2025; IBM Corp., Armonk, New York, United States). All tests were two-tailed, and significance was set at <0.05.

## Results

Baseline characteristics

In the patient cohort of 50 individuals, the median age was 11.5 years (range 4-25 years). The most common index and target joint was the knee joint. Only four out of the 50 patients (8%) were on secondary factor replacement prophylaxis, while the rest were on on-demand factor replacement therapy. None of the patients were on emicizumab prophylaxis. The median number of exposure days (number of days in a year requiring factor replacement for on-demand patients) was eight (range 2-156 days). The median ABR was six (range 2-16). The mean HJHS score was 8.68 (Standard error of mean or SEM ± 6.94; range 0-29) and the mean FISH score was 19.6 (SEM ±6.94; range 8-28). Mean VEGF level was 85.11 ± 9.01 pg/ml, while mean CRP was 1032.58 ± 242.88 pg/ml. For the 50 controls, mean VEGF was 56.9 ± 17.1 pg/ml and mean CRP was 140.26 ± 37.24 pg/ml (Table 1).

**Table 1:** Baseline patient characteristics ABR: annual bleed rate; SEM: standard error of mean; HJHS: Haemophilia joint health score; FISH: Functional independence score in haemophilia; VEGF: Vascular endothelial growth factor; CRP: C-reactive protein.

Parameters	Patients	Controls
Number	50	50
Median age (range)	11.5 years (4-25 years)	11.5 years (4-25 years)
Median age at diagnosis (range)	1 year (0.2 to 3.0 years)	-
Commonest index joint	Knee	-
Commonest target joint	Knee	-
Median ABR	6 (2-16)	-
Mean HJHS score (±SEM; range)	8.68 (±6.94; 0-29)	-
Mean FISH score (±SEM; range)	19.6 (±6.44, 8-28)	-
Mean VEGF level (pg/ml); SEM	85.11 ± 9.01	56.9 ± 17.1
Mean CRP level (pg/ml); SEM	1032.58 ± 242.88	140.26 ± 37.24

Comparative biomarker levels between cases and controls

To compare the biomarker means between cases and controls, Mann-Whitney test was applied. For VEGF levels, the difference of means was statistically different (U=250, P<0.001). Higher levels of VEGF were found in patients as compared to the controls. Similar results were found for the difference in mean CRP levels (U=270, two-tailed p value<0.001; Figure 1).

**Figure 1:**
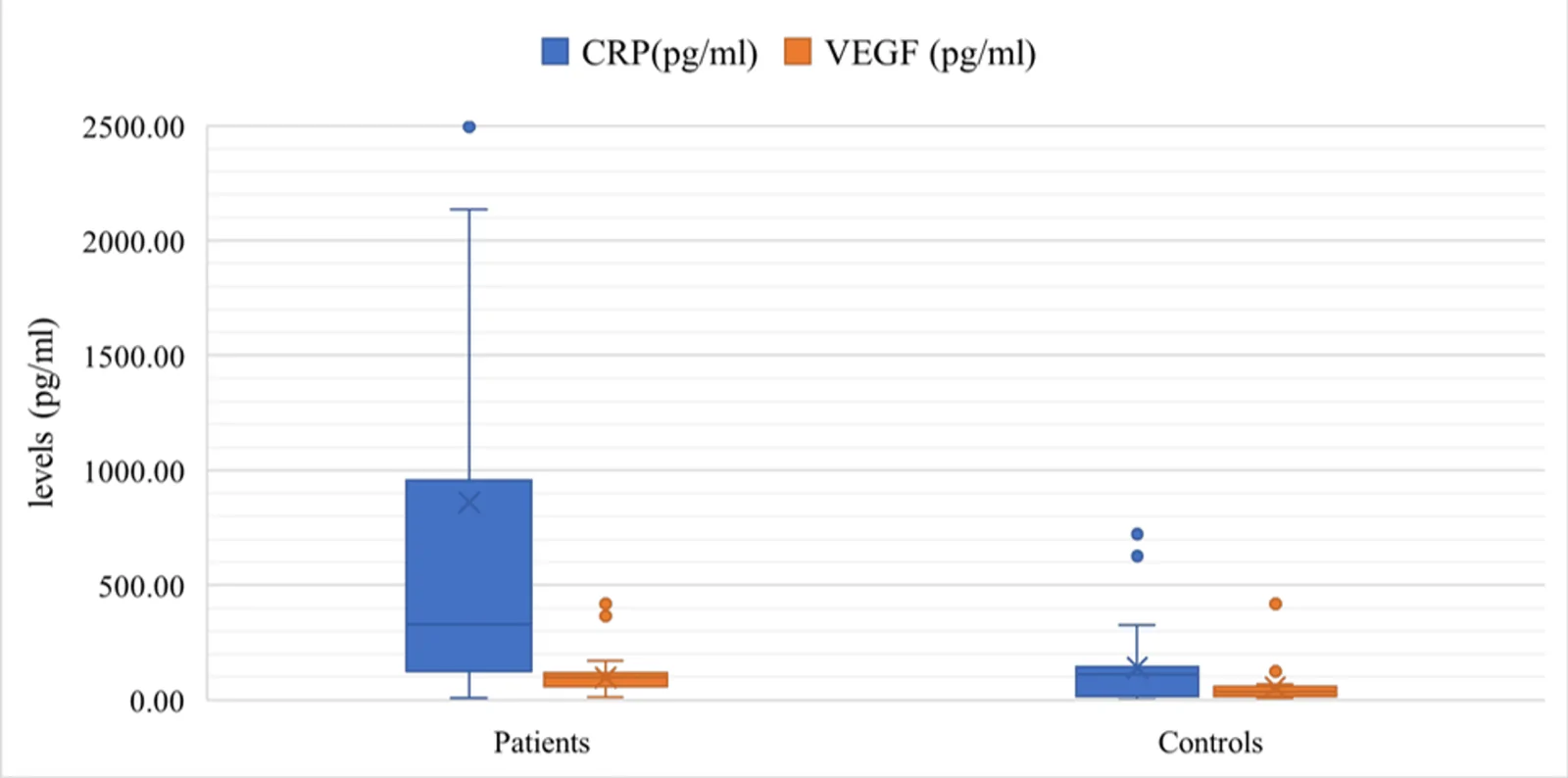
Comparison of VEGF and CRP levels between patients and controls for VEGF and CRP VEGF: Vascular endothelial growth factor; CRP: C-reactive protein.

Determination of VEGF and CRP cut-off between cases and controls

A ROC curve analysis was done to determine cut-offs between cases and controls. AUC for VEGF and CRP was 0.792 and 0.775 respectively, meaning that the tests had fair discriminant value. Keeping a sensitivity of 72% and a specificity of 87.5%, the VEGF cutoff which reasonably distinguished cases and controls was 68.85 pg/ml (Youden index: 0.595 - good test). For CRP levels, at a sensitivity of 64% and a specificity of 87.5%, a CRP level of 210.03 pg/ml provided a fair discriminant value (Youden index: 0.515 - fair test). Between VEGF and CRP, based on the Youden index and the AUC, VEGF was better in distinguishing cases and controls than CRP (Figure 2 and Table 2).

**Figure 2:**
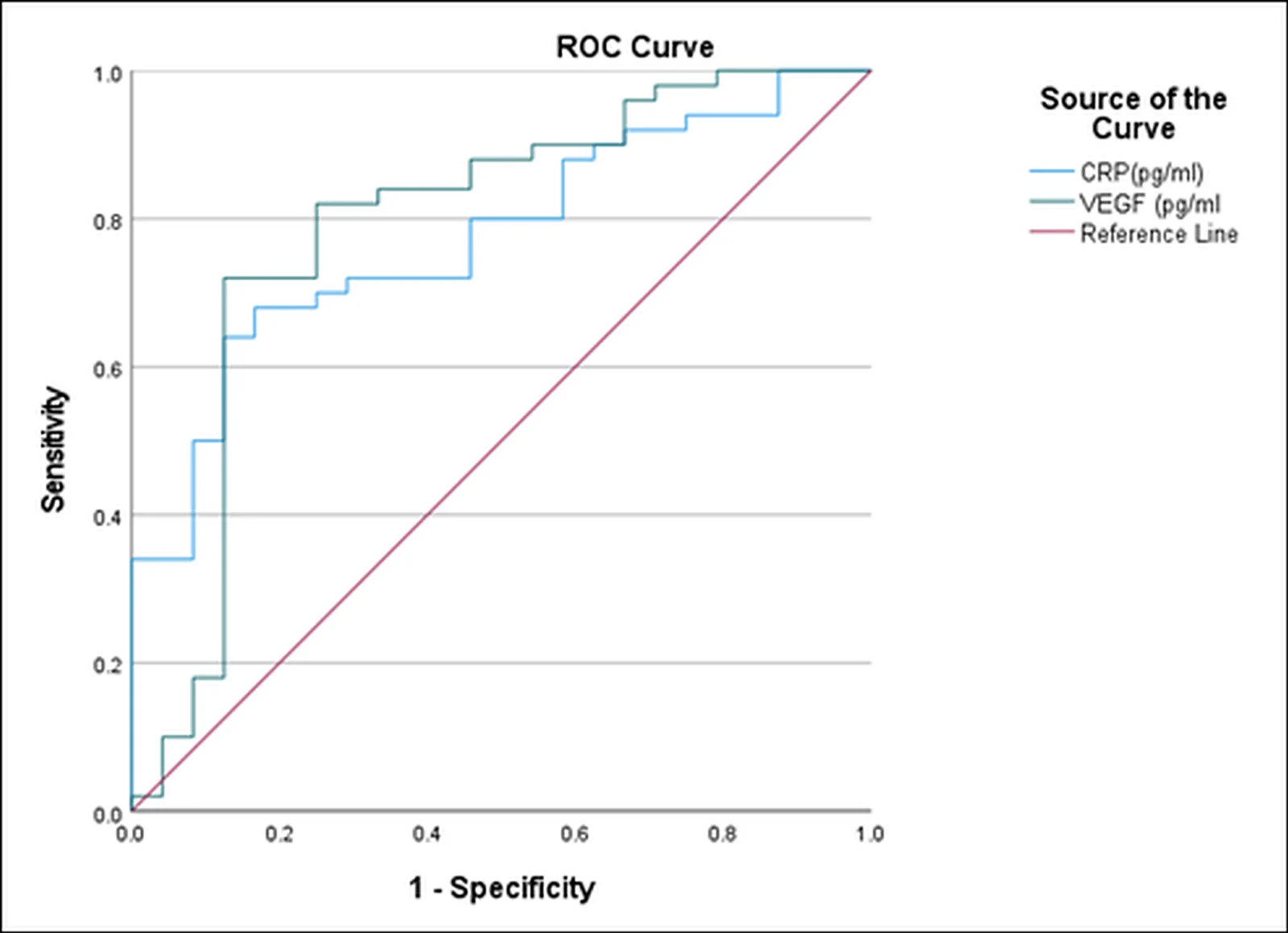
ROC curve analysis for finding the best cutoff value for VEGF and CRP ROC: Receiver operator characteristic; VEGF: Vascular endothelial growth factor; CRP: C-reactive protein.

**Table 2:** ROC analysis for finding the best cutoff value for VEGF and CRP ROC: Receiver operating characteristic; CI: confidence interval; VEGF: Vascular endothelial growth factor; CRP: C-reactive protein.

Parameter	Area under the curve (AUC)	Std. Error	95% Confidence Interval		Asymptotic Sig. (p value)
			Lower Bound	Upper Bound	
CRP (pg/ml)	0.775	0.054	0.668	0.882	<0.001
VEGF(pg/ml)	0.792	0.063	0.668	0.915	<0.001

Association between serum biomarkers and indices of joint health

Spearman’s rho and Kendall’s tau tests were used to determine correlations between VEGF/CRP levels and HJHS, FISH scores or ABR. There was statistically significant correlation (rho = -0.379, p<0.007; moderately strong inverse correlation) between FISH scores and VEGF levels, essentially meaning that patients with better functionality (higher FISH scores) had lower VEGF levels. There was significant correlation between CRP and FISH scores (rho= -0.364, p<0.009) and CRP and ABR scores (rho = 0.376, p<0.007; moderately strong positive correlation) respectively (Figure 3 and Table 3). 

**Figure 3:**
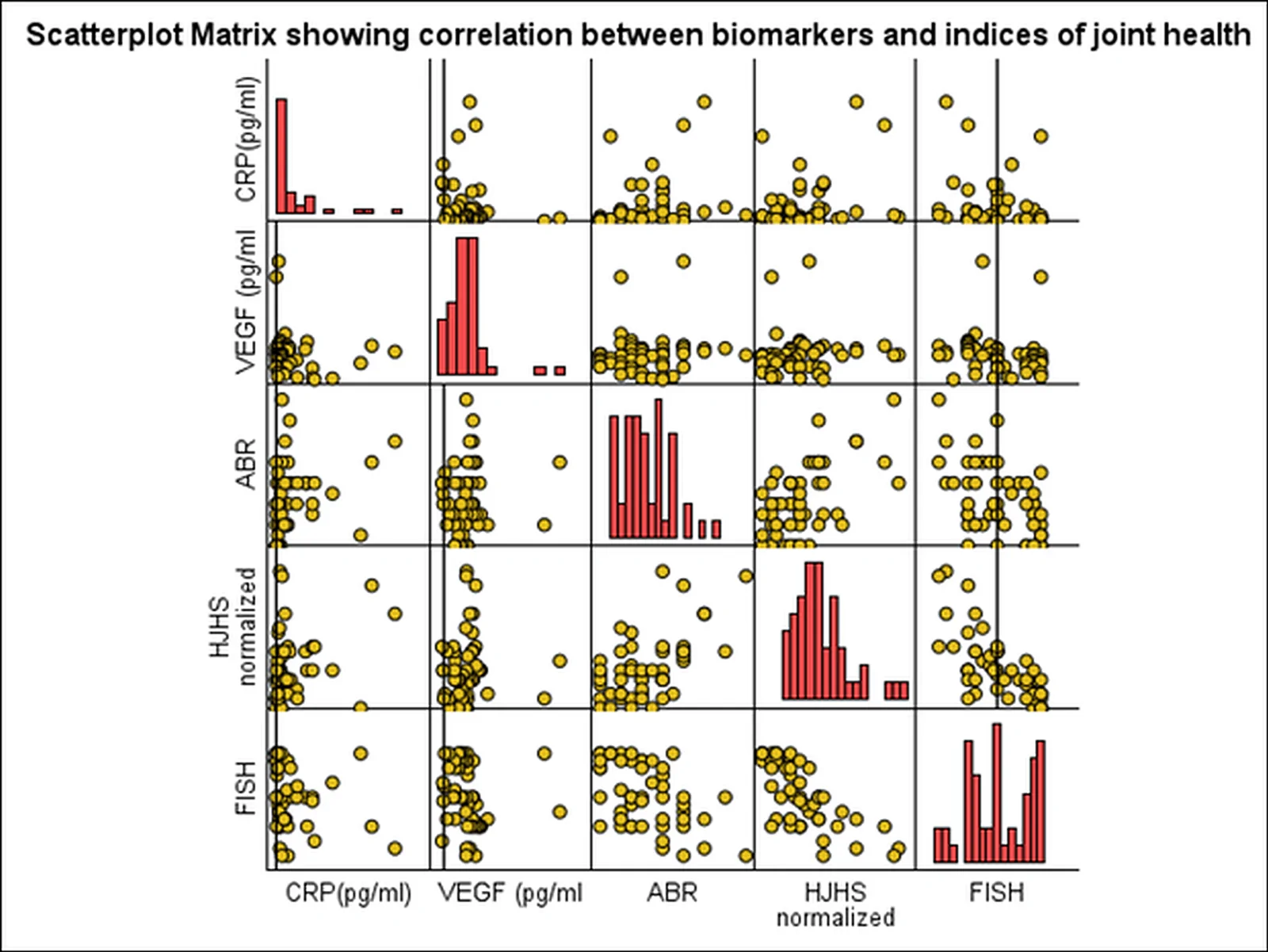
Correlations between CRP and VEGF with different indices of joint health CRP: C-reactive protein; VEGF: Vascular endothelial growth factor; ABR: Annual bleed rate, HJHS: Haemophilia joint health score; FISH: Functional independence score in haemophilia; CI: confidence interval.

**Table 3:** Correlations between CRP and VEGF with different indices of joint health CRP: C-reactive protein; VEGF: Vascular endothelial growth factor; ABR: Annual bleed rate, HJHS: Haemophilia joint health score; FISH: Functional independence score in haemophilia.

Correlations		Spearman's rho	Significance (2-tailed)	95% Confidence Intervals (2-tailed)	
				Lower	Upper
CRP	ABR	0.376	0.007	0.101	0.598
	HJHS	0.266	0.062	-0.022	0.513
	FISH	-0.364	0.009	-0.589	-0.087
VEGF	ABR	0.158	0.274	-0.135	0.425
	HJHS	0.204	0.156	-0.088	0.463
	FISH	-0.379	0.007	-0.600	-0.104

High CRP also correlated with the number of exposure days of factor replacement in the preceding year (rho=0.29; P<0.041). Similar results were obtained using Kendall’s tau test for correlation as well. VEGF had poor correlation with HJHS scores.

## Discussion

HA remains a major cause of morbidity in people with severe haemophilia A. Identifying simple serum biomarkers that reflect joint health and function could support earlier risk stratification [15] and more rational use of limited factor concentrate in these settings. The number of bleeds can affect the severity of joint damage, but inflammation and angiogenesis play a big role in this [19]. Some studies have shown that biomarkers could be used to detect bleeds even prior to imaging techniques like ultrasound and Magnetic Resonance Imaging (MRI) [14]. However, the determination of an accurate biomarker for HA comes with its own set of challenges and pitfalls [17]. In this exploratory cross‑sectional study from a haemophilia centre, we evaluated VEGF and CRP in relation to joint scores and functional status in predominantly on‑demand treated patients with severe haemophilia A.

We found that both VEGF and CRP were significantly higher in PWH compared to age‑matched male controls without arthropathy, consistent with prior reports [4,18] linking inflammatory and angiogenic mediators to haemophilic joint disease. The magnitude and direction of these differences are in line with larger series in which VEGF and CRP have been shown to be elevated in severe haemophilia [4,7,16,19]. ROC analysis demonstrated fair discriminatory performance for both biomarkers, with VEGF showing slightly higher AUC and Youden index, and cut‑offs comparable to those reported in paediatric and mixed cohorts [4,16,19]. These data support the concept that VEGF and CRP capture relevant biological differences between patients with severe haemophilia and healthy individuals.

Clinical examination of the musculoskeletal system plays an integral component of regular assessment and follow-up of PWH. Among the clinical systems available to assess joint health [20], HJHS is one of the most widely used [10,20]. In our study, the mean HJHS score was 8.68±6.94, whereas that recorded by Saboony et al. [14] was 17.03±7.005, and Andrawes et al. [19], i.e., 4.5±3.0, had a wide range of variation. However, both these studies could demonstrate a strong positive correlation between serum VEGF levels and HJHS score, which contrasts with our study where HJHS did not show a correlation with either VEGF or CRP levels.

The mean FISH score was 19.6 (SD ±6.94; range 8-28) and we found strong correlations between FISH score and both VEGF and CRP levels. Saboony et al. found total FISH score was 30.7±4 and discovered a non-significant negative correlation with VEGF-A [14]. Most of their patients were on regular factor replacement and this might account for the higher FISH scores they reported. One possible explanation is that in real‑world practice, functional scores may be more sensitive to the cumulative impact of recurrent bleeds, pain and muscle weakness than a single time‑point joint examination, particularly in patients with heterogeneous ages and treatment exposures.

In our study, we assessed the ABR of the patients and found a significant correlation of CRP with ABR, but not with VEGF. None of the previous studies have shown a correlation of either CRP or VEGF with ABR and so it was not possible to compare our findings.

We did an ROC curve analysis in our study which revealed AUC for VEGF and CRP was 0.792 and 0.775, respectively. We found a cutoff value of 68.85 pg/mL for VEGF levels with sensitivity of 72% and specificity of 87.5%. Saboony et al. [14] reported a cut-off value of more than 65 ng/L with a sensitivity of 83.3% and specificity of 70%. The sensitivities and specificities of VEGF-A in detecting HA were 78% and 88%, respectively in the study by Andrawes et al. [19]. Xu et al. [4] reported that the ROC curve produced a sensitivity of 82.8% and a specificity of 68.3% for VEGF, which was also close to our results. For CRP, with sensitivity of 64% and a specificity of 87.5%, a level of 210.03 pg/ml provided a reasonably good cutoff for cases vs controls; however, we found serum VEGF to be the better marker to distinguish between patients with haemophilia with joint bleeds vs healthy controls.

There are various limitations to our study. The small sample size does not allow generalisation of our findings. Being a cross sectional study, the results cannot help us determine whether VEGF and/or CRP levels are helpful in predicting the recurrence of bleeds. Thirdly, we did not correlate the serum levels with imaging modalities like MRI which are gold standard of assessing joint health due to logistic constraints and lastly, some patients were on prophylaxis while most were on-demand therapy. That may lead to confounding effects.

Longitudinal follow up and serial assessment of these markers in the same patients may help in predicting and detecting bleeds early, generating predictive models that may guide prophylaxis and on-demand therapy. Suitable biomarkers can also be an area of drug development for prophylaxis and treatment.

## Conclusions

From the results of this study, we can confirm that VEGF and CRP levels are increased in the serum of patients with severe haemophilia A and correlate significantly with FISH. Further longitudinal studies are needed to validate these findings and determine the utility of these biomarkers in evaluating joint health status.
